# TRAF6 Inhibition Rescues Dexamethasone-Induced Muscle Atrophy

**DOI:** 10.3390/ijms150611126

**Published:** 2014-06-20

**Authors:** Hualin Sun, Yanpei Gong, Jiaying Qiu, Yanfei Chen, Fei Ding, Qing Zhao

**Affiliations:** 1 Jiangsu Key Laboratory of Neuroregeneration, Co-Innovation Center of Neuroregeneration, Nantong University, Nantong 226001, Jiangsu, China; E-Mails: sunhl@ntu.edu.cn (H.S.); ntgongyp@sohu.com (Y.G.); scudlcms@163.com (J.Q.); chenq_js@163.com (Y.C.); 2 The Orthopedic Institute of First Affiliated Hospital, General Hospital of Chinese PLA, Beijing 100048, China

**Keywords:** TRAF6, muscle atrophy, glucocorticoid

## Abstract

Tumor necrosis factor receptor-associated factor 6 (TRAF6), a unique E3 ubiquitin ligase and adaptor protein, is involved in activation of various signaling cascades. Recent studies identify TRAF6 as one of the novel regulators of skeletal muscle atrophy. The role of TRAF6 in glucocorticoid-induced muscle atrophy, however, remains to be elucidated. In this study, we show that TRAF6 and its downstream signaling molecules, muscle atrophy F-box (MAFBx) and muscle ring finger 1 (MuRF1), were all upregulated in dexamethasone-induced atrophy of mouse C2C12 myotubes or mouse tibialis anterior (TA) muscle. To further investigate the role of TRAF6 in dexamethasone-induced muscle atrophy, TRAF6-siRNA was used to transfect cultured C2C12 myotubes or was injected into the TA muscle of mice respectively, and we note that TRAF6 knockdown attenuated dexamethasone-induced muscle atrophy *in vitro* and *in vivo*, and concomitantly decreased the expression of MuRF1 and MAFBx. Our findings suggest that a decreased expression of TRAF6 could rescue dexamethasone-induced skeletal muscle atrophy through, at least in part, regulation of the expression of MAFBx and MuRF1.

## 1. Introduction

Skeletal muscle atrophy, resulting from increased myofibrillar protein breakdown, is a complex biochemical process occurring under various pathophysiological conditions, such as aging, disuse, starvation, severe injury, sepsis, cancer and other cachectic diseases [1,2,3,4]. Glucocorticoids are the most widely used anti-inflammatory drugs, but their prolonged use is also likely to cause muscle atrophy by inhibiting amino acid transport into the muscle, suppressing muscle protein synthesis, and stimulating muscle protein degradation through up-regulation of the ubiquitin ligases, including muscle atrophy F-box (MAFbx; also called atrogin-1) and muscle ring finger 1 (MuRF1) [3,5,6,7,8,9]. Although a number of explanations have been proposed [5,7,10,11,12], the precise molecular mechanisms by which glucocorticoids induce muscle atrophy are not well understood. Muscle atrophy often worsens the quality of life for patients, but effective countermeasures are still lacking to help patients recover from different types of muscle atrophy. In consequence, considerable attention has been focused on the understanding of molecular mechanisms responsible for dexamethasone-induced muscle atrophy and the development of novel therapeutic strategies.

Tumor necrosis factor (TNF) receptor associated factor 6 (TRAF6) is a member of TRAF family, and it functions as a crucial signaling molecule to regulate a diverse array of physiological processes, including innate immunity, adaptive immunity, bone metabolism, and the development of mammary glands, lymph nodes, skin and the central nervous system [13]. Among TRAF family members, TRAF6 has unique properties, which enable it to not only mediate tumor necrosis factor receptor (TNFR) family signaling, but also to affect signaling downstream of an unrelated family of receptors, the interleukin-1 (IL-1) receptor/Toll-like receptor (IL-1R/TLR) superfamily [14]. TRAF6 is also an important E3 ubiquitin ligase, and works with the dimeric ubiquitin-conjugating enzyme Ubc13/Uev1A to promote the unique Lys-63-linked poly-ubiquitin chains, rather than the conventional Lys-48-linked poly-ubiquitin chains that target proteins for degradation [15]. TRAF6 was proven to be important for the activation of many signaling pathways including NF-κB, MAPK, and PI3K/Akt in response to various cytokines [16], and for the interaction with multiple components of the ubiquitin-proteasome system (UPS) in some cell types [17,18,19]. In this way, TRAF6 regulates skeletal muscle mass and the UPS activation in denervation or starvation-induced muscle atrophy [15,16,20].

We hypothesized that glucocorticoid-induced muscle atrophy might be linked to the expression and regulation of *TRAF6*. To test the hypothesis, in this study, we examined the expression of *TRAF6* in dexamethasone-induced muscle atrophy under *in vivo* and *in vitro* conditions, and further investigated the regulating effect of *TRAF6* on myotube/muscle atrophy using knockdown experiments.

## 2. Results

### 2.1. Up-Regulation of Tumor Necrosis Factor Receptor-Associated Factor 6 (TRAF6), Muscle Atrophy F-Box (MAFBx), and Muscle Ring Finger 1 (MuRF1) in Muscle and Myotube Treated with Dex

**Figure 1:**
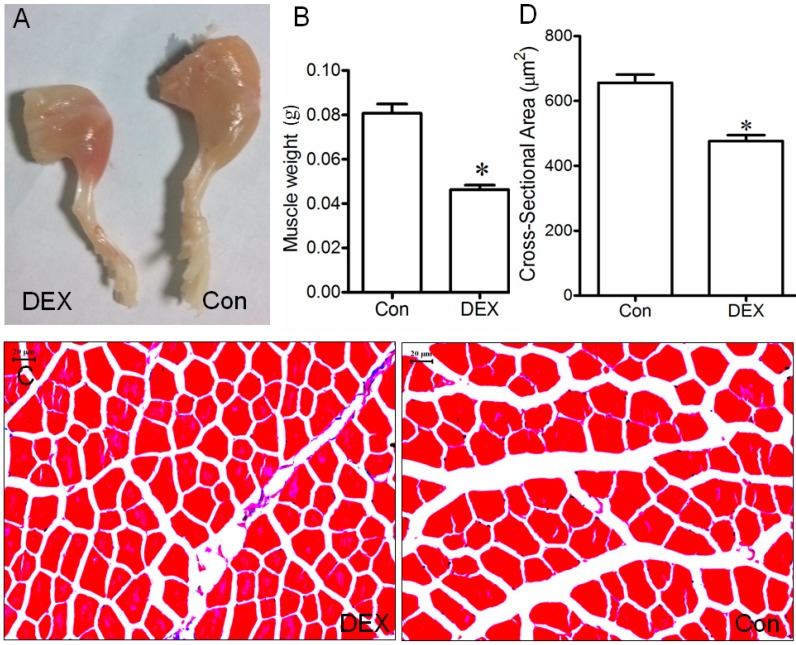
(**A**) Image of the hind limb of mice receiving daily intraperitoneal injection with 0.1 mL of vehicle (saline, control) or Dex (dexamethasone sodium phosphate in saline, 10 mg/kg) for 14 days, respectively; (**B**,**D**) Bar graphs showed the weight (**B**) and cross sectional area (CSA, **D**) of the TA muscle of mice injected with vehicle (control) or Dex respectively. Data are presented as mean ± SD, *n* = 9 per animal group, * *p* < 0.05 *versus* control; Also shown (**C**) is a representative image of Masson trichrome staining for determining the CSA of the mouse TA muscle. Scale bar = 20 μm.

Treatment of mice with 10 mg/kg Dex (dexamethasone) induced a significant decrease in either the weight or cross-sectional area (CSA) of the tibialis anterior (TA) muscle compared to treatment of mice with vehicle (Figure 1), confirming that an animal model of Dex-induced muscle atrophy was successfully constructed in this study.

**Figure 2:**
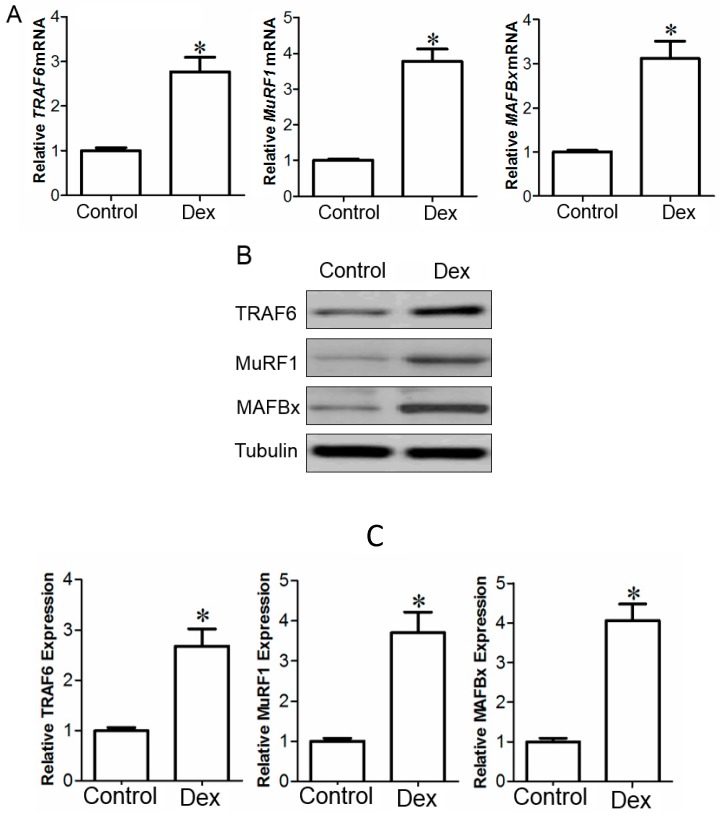
The qPCR (quantitative real-time PCR) (**A**) and Western blot analysis (**B**,**C**) showed the mRNA (**A**) and protein (**B**,**C**) expressions of *TRAF6*, *MAFBx*, and *MuRF1* in the TA muscle of mice injected with vehicle (saline, control) or Dex (dexamethasone sodium phosphate in saline, 10 mg/kg) respectively. Data are presented as mean ± SD, *n* = 9 per animal group, * *p* < 0.05 *versus* control. Also shown (**B**) is a representative Western blot image. *GAPDH* and tubulin were used as a loading control in qPCR and Western blot analysis.

The expression of *TRAF6*, *MAFBx*, and *MuRF1* at the mRNA and protein levels in TA muscles of mice treated with 10 mg/kg Dex was significantly increased compared to that treated with vehicle, respectively, as determined by qPCR (quantitative real-time PCR) and Western blot analysis, respectively (Figure 2).

**Figure 3:**
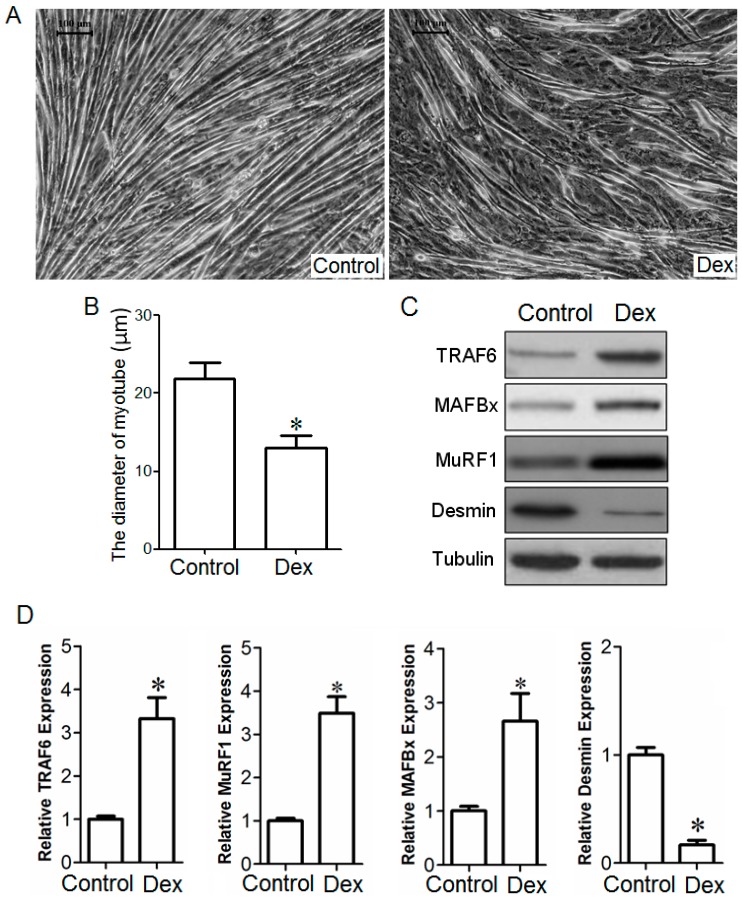
(**A**) Micrograph showed the morphology of C2C12 myotubes cultured in vehicle (0.1% ethanol-containing plain medium, control) or stimulated by Dex (dexamethasone in vehicle) respectively. Scale bar = 100 μm; (**B**) Bar graph compared the diameter of C2C12 myotubes cultured in vehicle (control) or stimulated by Dex respectively; and (**C**,**D**) Representative Western blot image and Bar graphs displayed the protein expression of TRAF6, MAFBx, MuRF1, and desmin in C2C12 myotubes cultured in vehicle (control) or stimulated by Dex respectively. Tubulin were used as a loading control in Western blot analysis. All data in bar graphs are presented as mean ± SEM (standard error of the mean) from three independent experiments, * *p* < 0.05 *versus* control.

During differentiation of C2C12 cells, C2C12 myoblasts fused together to form myotubes. After the formed C2C12 myotubes were stimulated with 100 µM Dex for 48 h, microscopic observation showed that the myotubes were atrophied (Figure 3A), while quantitative measurement indicated that the myotube diameter in C2C12 myotubes stimulated by Dex was significantly lower than that in C2C12 myotubes cultured in vehicle control (Figure 3B,C). Concomitantly, Western blot analysis indicated that the protein expression of TRAF6, MAFBx, or MuRF1 was higher in Dex-stimulated C2C12 myotubes than in C2C12 myotubes cultured in vehicle (0.1% ethanol-containing plain medium). In addition, the protein expression of desmin in Dex-treated C2C12 myotubes was expectedly decreased compared to that in C2C12 myotubes cultured in vehicle because of the atrophy-inducing effect of Dex (Figure 3C,D).

### 2.2. Effect of TRAF6 Knockdown on Dex-Induced Myotube Atrophy in Vitro

**Figure 4:**
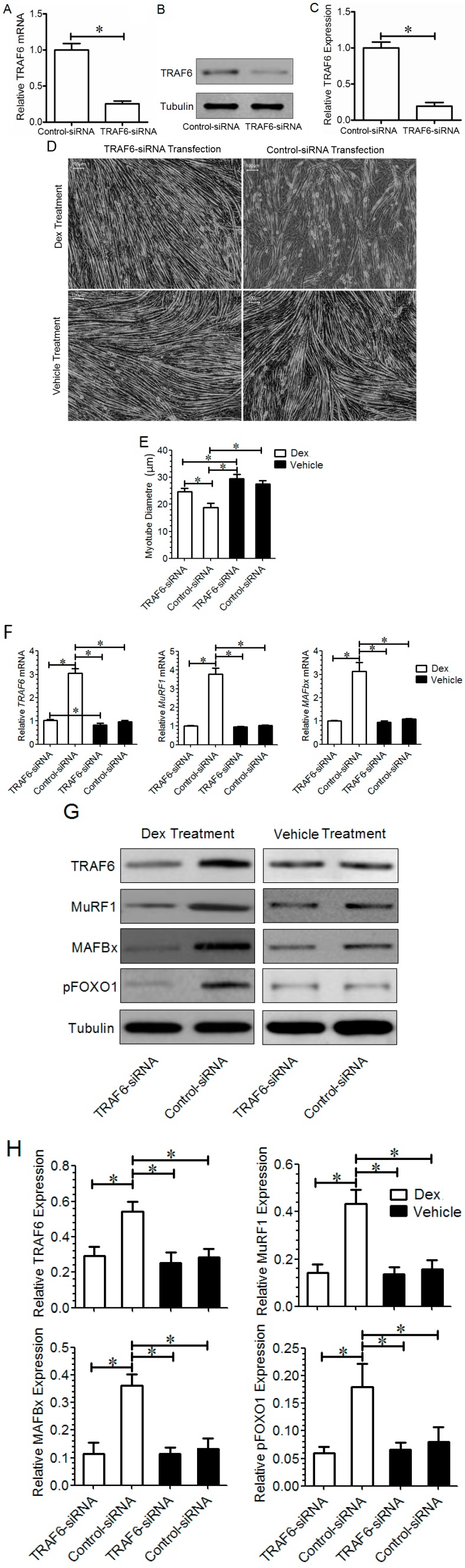
The qPCR (**A**) and Western blot analysis (**B**,**C**) showed that C2C12 myotubes were transfected with *TRAF6*-siRNA or control-siRNA; Micrographs (**D**) showed the morphology of Dex- or vehicle-treated C2C12 myotubes after transfection with *TRAF6*-siRNA and control-siRNA respectively. Scale bar = 100 μm; Bar graph (**E**) showed the diameter of Dex- or vehicle-treated C2C12 myotubes after transfection with *TRAF6*-siRNA and control-siRNA respectively; The qPCR and Western blot analysis showed the mRNA (**F**) and protein (**G**,**H**) expressions of *TRAF6*, *MAFBx*, and *MuRF1*, as well as the protein expression of pFOXO-1 in Dex- or vehicle-treated C2C12 myotubes after transfection with *TRAF6*-siRNA and control-siRNA respectively. *GAPDH* and tubulin were used as a loading control in qPCR and Western blot analysis. All data in bar graphs are presented as mean ± SEM from three independent experiments, * *p* < 0.05.

The qPCR and Western blot analysis confirmed that C2C12 myotubes were successfully transfected with *TRAF6*-siRNA (Figure 4A–C). Microscopic observation showed that *TRAF6* knockdown attenuated Dex-induced atrophy in C2C12 myotubes, and quantitative comparison indicated that the diameter of C2C12 myotubes transfected with *TRAF6*-siRNA was significantly larger than that of C2C12 myotubes transfected with control-siRNA (Figure 4D,E). We also examined the effects of *TRAF6* knockdown on vehicle-treated C2C12 myotubes, and noted that ablation of *TRAF6* did not induce significant hypertrophy in C2C12 myotubes (Figure 4D,E). Interestingly, the mRNA and protein expressions of *TRAF6*, *MAFBx*, and *MuRF1*, as well as the protein expression of phosphorylated FOXO-1 (pFOXO-1) in Dex-treated C2C12 myotubes were significantly decreased after transfection with *TRAF6*-siRNA compared to that after transfection with control-siRNA. In contrast, the mRNA and protein expressions of *TRAF6*, *MAFBx*, and *MuRF1*, as well as the protein expression of pFOXO-1 in vehicle-treated C2C12 myotubes after transfection with *TRAF6*-siRNA were not significantly different from those after transfection with control-siRNA (Figure 4F–H). All the results suggested that *TRAF6* knockdown alleviated Dex-induced up-regulation of *TRAF6* downstream molecules in C2C12 myotubes.

### 2.3. Effect of TRAF6 Knockdown on Dex-Induced Muscle Atrophy in Vivo

**Figure 5:**
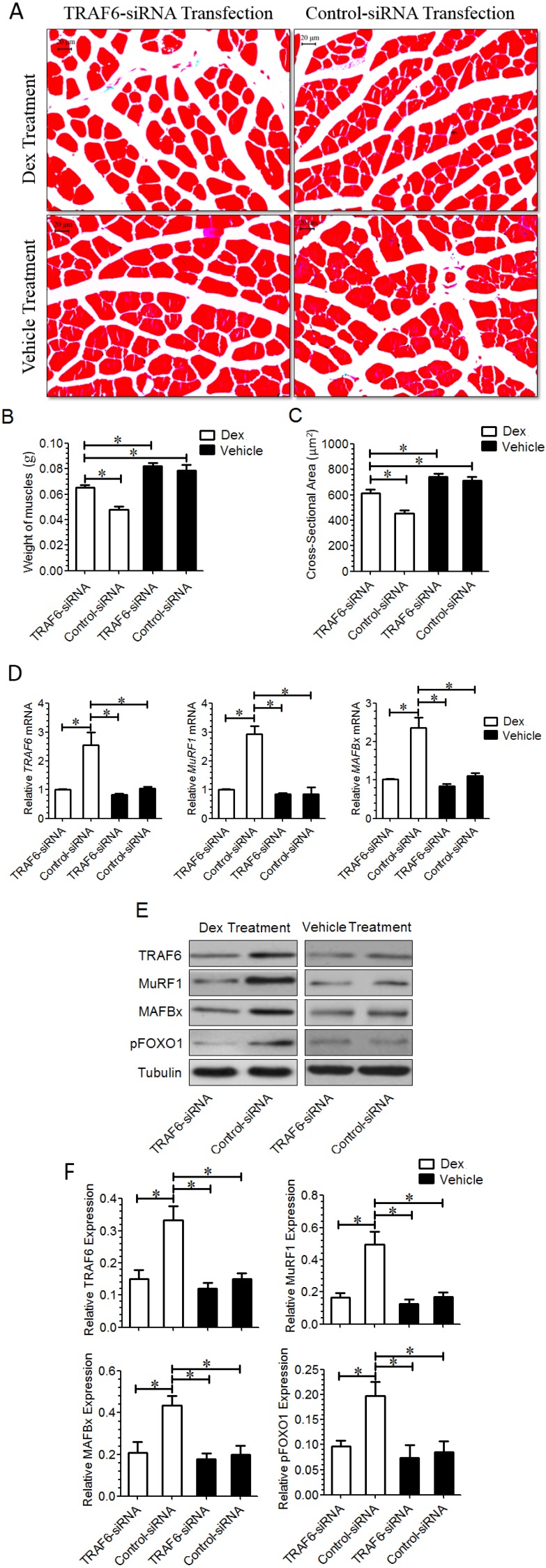
(**A**) Masson trichrome staining image of the TA muscle from mice co-injected with both Dex and control-siRNA, with both Dex and *TRAF6*-siRNA, with both vehicle and control-siRNA, or with both vehicle and *TRAF6*-siRNA, respectively, for 14 days; (**B**,**C**) Bar graphs showed the weight (**B**) and cross sectional area (CSA, **C**) of the TA muscle from mice co-injected with each of the above four combinations respectively. Scale bar =20 μm; (**D**–**F**) The qPCR and Western blot analysis showed the comparison in the mRNA (**D**) and protein; and (**E**,**F**) expression of TRAF6, MAFBx, and MuRF1, as well as the protein expression of pFOXO-1 in the TA muscle of mice co-injected with each of the above four combinations respectively. *GAPDH* and tubulin were used as a loading control in qPCR and Western blot analysis, respectively. All data in bar graphs are presented as mean ± SD, *n* = 9 per animal group,* *p* < 0.05.

Microscopic observation showed that *TRAF6* knockdown reduced Dex-induced muscle atrophy in animals, and quantitative comparison further indicated that the weight or the cross-sectional area (CSA) of the TA muscle from mice injected with *TRAF6*-siRNA was significantly larger than that from mice injected with control-siRNA. Meanwhile, we noted that transfection with *TRAF6*-siRNA and with control-siRNA did not induce significant difference in the morphology of the TA muscle from mice injected with vehicle (Figure 5A–C). The qPCR and Western blot analysis demonstrated that the mRNA and protein expressions of *TRAF6*, *MAFBx*, and *MuRF1*, as well as the protein expression of pFOXO-1 were significantly lower in the TA muscle from mice co-injected with Dex and *TRAF6*-siRNA than in the TA muscle from mice co-injected with Dex and control-siRNA (Figure 5D–F), suggesting that *TRAF6* knockdown alleviated Dex-induced up-regulation of *TRAF6* downstream molecules in the TA muscle of mice. In contrast, the expression of *TRAF6* downstream molecules in the TA muscle of mice co-injected with vehicle and *TRAF6*-siRNA was not significantly different from that co-injected with vehicle and control-siRNA (Figure 5D–F), suggesting that *TRAF6* knockdown failed to induce muscle hypertrophy.

## 3. Discussion

This study for the first time identifies *TRAF6* as a novel regulator of Dex-induced muscle atrophy. Our results indicate that Dex induces atrophy of mouse TA muscles or mouse C2C12 myotubes, and concomitantly up-regulates the expression of *TRAF6* in the *in vivo* and *in vitro* models of muscle atrophy. Our finding is consistent with previous studies that showed that *TRAF6* expression is significantly up-regulated in denervation- or starvation-induced muscle atrophy [15,16]. Previous studies also reveal that *TRAF6* affects denervation- or starvation-induced muscle atrophy through regulation of muscle-specific ubiquitin ligases *MuRF1* and *MAFBx*, while the E3 ubiquitin ligase activity of TRAF6 is essential for starvation-induced muscle atrophy [15,16]. Inspired by these observations, we tried to determine whether MuRF1 and MAFBx could also function as the downstream signaling molecules of TRAF6 in Dex-induced muscle atrophy. Our results show that both *MuRF1* and *MAFBx* were up-regulated in Dex-induced muscle atrophy under *in vivo* and *in vitro* conditions.

To further test the involvement of *TRAF6* in Dex-induced muscle atrophy, we examined the impact of *TRAF6* knockdown on C2C12 myotubes or TA muscles that had been treated with Dex, and found that *TRAF6* inhibition could attenuate Dex-induced muscle atrophy *in vitro* and *in vivo*. This result is consistent with a previous finding that *TRAF6* inhibition reduced denervation-induced muscle atrophy [15]. Meanwhile, it was also noted that targeted ablation of *TRAF6* suppressed the expression of MuRF1 and MAFBx as well as pFOXO-1, suggesting that TRAF6 might exert its function through, at least in part, the regulation of muscle-specific ubiquitin ligases in Dex-induced muscle atrophy.

In view of the above observations, *TRAF6* was upregulated in Dex-induced muscle atrophy *in vitro* and *in vivo*. Targeted ablation of *TRAF6* could reduce Dex-induced muscle atrophy *in vitro* and *in vivo*. In addition, we inferred that TRAF6 exerted its function through, at least in part, the regulation of muscle-specific ubiquitin ligases (*MuRF1* and *MAFBx*) in Dex-induced muscle atrophy. However, further research is still needed to provide a good understanding of the function and mechanism of *TRAF6* in muscle atrophy.

## 4. Experimental Section

### 4.1. Animal Care and Treatments

Animal experiments were carried out in accordance with the institutional animal care guidelines and approved by the administration Committee of Experimental Animals, Jiangsu, China.

Male ICR mice with similar initial body weights (about 20 g) were routinely maintained under the same conditions (temperature 22 °C, 12:12 h light-dark cycle) with free access to standard laboratory rodent chow and water. For animal treatments, mice were randomly divided into two groups to receive daily intraperitoneal injection with 0.1 mL of vehicle (saline) and Dex (dexamethasone sodium phosphate in saline, 10 mg/kg) (Shenyang Everbright Pharmaceutical Co., Ltd, Shenyang, China) [21], respectively. Transfection with TRAF6-siRNA or control-siRNA was initiated on the same day, as described previously [22]. In brief, mice were anesthetized and the TA muscle was injected with TRAF6-siRNA (5 nmol) or control-siRNA (5 nmol) every three days for 2 weeks. TRAF6-siRNA and control-siRNA were purchased from RiboBio (Guangzhou, China). All animals were killed 14 days after the beginning of transfection.

After the mice were killed, the TA muscle samples were excised from two legs of animals, immediately weighed, frozen on liquid nitrogen, and stored at −80 °C for subsequent RNA and protein extractions. Total RNAs were extracted with the TRIzol Reagent (Invitrogen, Carlsbad, CA, USA), and the RNA quality was assessed on an Agilent 2100 Bioanalyzer (Agilent Technologies, Palo Alto, CA, USA). Total proteins were extracted using RIPA buffer (50 mM Tris-HCl pH 7.4, 150 mM NaCl, 5 mM EDTA, 1% Nonidet P-40, 1% sodium deoxycholate, 0.1% SDS, 1% aprotinin, 50 mM NaF, 0.1 mM Na_3_VO_4_), and quantified with the Bio-Rad protein assay kit (Bio-Rad, Hercules, CA, USA). The CSA of muscle fiber was evaluated in 5-µm sections of TA muscle processed with Masson trichrome staining. In brief, the paraffin embedded tissue sections were deparaffinized, dehydrated, and placed in Harris hematoxylin (Sigma-Aldrich, St. Louis, MO, USA) for 10 min at room temperature, then stained with Biebrich scarlet-acid fuchsin (Sigma-Aldrich, Milwaukee, WI, USA) for 30 min and with aniline blue for 15 s in turn. Afterwards, the sections were washed, dried, dehydrated, and photographed under a microscope (Leica Microsystems, Wetzlar, Germany). The analysis of the CSA of muscle fibers was done by two researchers in a blind manner.

### 4.2. Cell Culture and Transfection

Cell culture was performed as described previously [8,23]. Briefly, C2C12 cells were obtained from the Cell Bank at the Chinese Academy of Sciences (Shanghai, China). They were cultured in high-glucose DMEM (Gibco-BRL, Gaithersburg, MD, USA) supplemented with 10% FBS (Gibco-BRL, Gaithersburg, MD, USA), 100 μg/mL of streptomycin (Sigma-Aldrich, St. Louis, MO, USA), and 100 U/mL of penicillin (Sigma-Aldrich, St. Louis, MO, USA) in a 10% CO_2_ humidified atmosphere at 37 °C. C2C12 cells that had grown to approximately 80% confluence in culture flasks were trypsinized, and seeded into 6-well culture plates to allow incubation in DMEM containing 10% FBS until reaching about 90% confluence. After the medium was replaced with DMEM containing 2% horse serum, the cells were induced to differentiate into myotubes until fusion of more than 90% cells into myotubes. After differentiation for 7 days, the formed C2C12 myotubes were treated with 100 nM Dex in 0.1% ethanol for 48 h, and collected for RNA and protein preparation. The C2C12 myotubes cultured in vehicle (0.1% ethanol-containing medium) were used as control.

Transfections were carried out using riboFECT™ CP reagent (RiboBio, Guangzhou, China) according to the manufacturer’s instructions. 100 nM of *TRAF6*-siRNA or control-siRNA (RiboBio) were transfected into C2C12 myotubes, respectively. 6 h later, the C2C12 myotubes were treated with 100 nM Dex in vehicle (0.1% ethanol-containing plain medium) or vehicle for 48 h and collected for RNA and protein preparation with the aforementioned procedures.

After treatments, transfected C2C12 myotubes were photographed under a phase contrast microscope (Leica Microsystems, Wetzlar, Germany) by researchers without knowledge of treatment. Two diagonal lines were drawn across each image. The diameter of myotubes was measured where the diagonal lines transected myotubes from 6 random fields by using through Image-Pro Plus software (Media Cybernetics, Silver Springs, MD, USA).

### 4.3. Real-Time Quantitative RT-PCR (qPCR)

The RNA samples were subjected to reverse transcription using a reverse transcription primer (Oligo(dt)). The cDNA was synthesized using an iScript cDNA synthesis kit (BioRad, Hercules, CA, USA) according to the kit manual. All primers were purchased from Shanghai Generay Biotech Co., Ltd. (Shanghai, China). The primers used in this study include: *TRAF6* F: GCAGAGGAATCACTTGGCACG, R: CACGGACGCAAAGCAAGGTT, *MuRF1* F: GTCATCCTGCCCTGCCAACA, R: CAACGGAAACGACCTCCAGAC, *MAFbx* F: CACATCCCTGAGTGGCATCG, R: CCAGCACAGACTTGCCGACT, *GAPDH* F: AAGGTCATCCATGACAACTTTGGC, R: ACAGTCTTCTGGGTGGCAGTGAT. The qPCR reactions were performed using the iTaq Fast SYBR Green Supermix (Bio-Rad, Hercules, CA, USA) exactly following the manufacturer’s instructions. Quantitative data of mRNA expressions were acquired and analyzed using an Applied Biosystems 7500 real-time PCR system (Applied Biosystems, Foster City, CA, USA). The cycle threshold (*C*_t_) values, corresponding to the number of PCR cycles at which fluorescence emission reached a threshold above baseline emission, were determined. The relative mRNA expression was measured through the 2^−ΔΔ*C*t^ method [24]. Glyceraldehyde-3-phosphate dehydrogenase (*GAPDH*) served as an internal control.

### 4.4. Western Blot Analysis

Western blot analysis was performed as described previously [25]. In brief, protein samples electrophoretically separated by SDS-PAGE, and then transferred to PVDF membranes, which were blocked with 5% nonfat dry milk in Tris-buffer saline (TBS), followed by incubation with primary antibodies: rabbit anti-TRAF6 polyclonal antibody (1:1000, ABGENT, San Diego, CA, USA), goat anti-MuRF1 polyclonal antibody (1:1000, R&D System, Minneapolis, MN, USA), anti-MAFbx polyclonal antibody (1:1000, LifeSpan Biosciences, Seattle, WA, USA), rabbit anti-beta tubulin polyclonal antibody (1:2000, Abcam, Cambridge, MA, USA), rabbit anti-desmin polyclonal antibody (1:2000, Abcam) and rabbit anti-pFOXO1 (1:1000, Cell Signaling Technology, Beverly, MA, USA) in TBST (TBS plus 0.1% Tween-20), at 4 °C overnight. Then, the membrane was probed with horseradish peroxidase-coupled mouse anti rabbit/goat IgG antibodies (AB Biotec, Stockholm, Sweden). The signal was detected using an ECL detection kit (Amersham, Buckinghamshire, UK) and the intensity of the bands was scanned by densitometry.

### 4.5. Statistical Analysis

All data are expressed as means ± SD or SEM as specially indicated. One-way ANOVA was used to compare differences between groups. All statistical analyses were conducted with a STATA 7.0 software package (Stata Corp., College Station, TX, USA). Significance levels were set at *p* < 0.05.

## 5. Conclusions

Our data showed that *TRAF6* and its downstream target genes, *MAFBx* and *MuRF1*, displayed a significant up-regulation in Dex-induced atrophy of mouse TA muscles or mouse C2C12 myotubes, suggesting that increased *TRAF6* expression might play an important role in Dex-induced muscle atrophy. Suppression of *TRAF6* expression in mouse TA muscle and C2C12 myotubes was achieved through the use of small interfering RNA. The qPCR and Western blot analysis showed that *TRAF6*-siRNA could successfully inhibit the expression of *TRAF6*, accompanied by a decreased expression of *MuRF1* and *MAFBx*, in mouse muscle and in C2C12 myotubes. Microscopic observation showed that *TRAF6* knockdown attenuated Dex-induced atrophy in the TA muscle and C2C12 myotubes. All our results suggested that a decreased expression of *TRAF6* could rescue Dex-induced skeletal muscle atrophy through, at least in part, regulation of the expression of *MAFBx* and *MuRF1*.
